# In This Issue: Volume 117, Issue 3, March 2026

**DOI:** 10.1111/cas.70345

**Published:** 2026-03-02

## Expression of the CXCR4 S338X Variant Improves Anti‐Leukemia Efficacy of Anti‐CD19 CAR‐T Cells

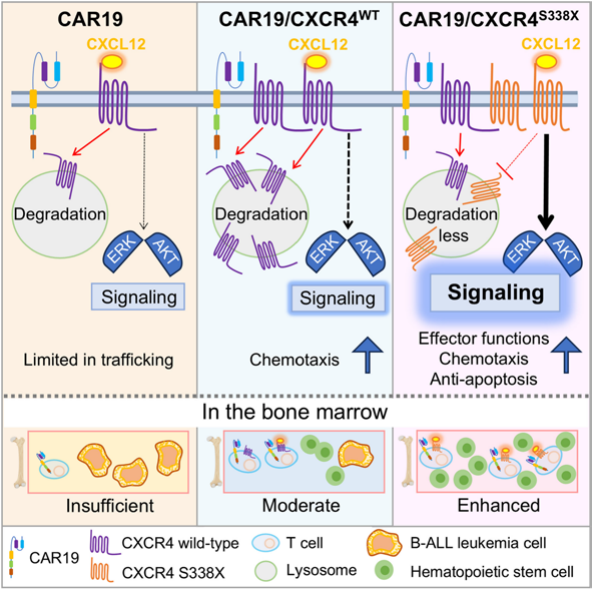

Treating B‐cell acute lymphoblastic leukemia (B‐ALL) is difficult, since immature B‐cell precursors become cancerous and drive the disease. These malignant cells share many surface marker proteins with healthy white blood cells. They also reside in the bone marrow, a protective microenvironment that limits immune cell access and supports leukemia cell survival, preventing immune cells from effectively killing cancerous cells.

Chimeric antigen receptor T cell (CAR‐T) therapy uses T cells with modified receptors to identify and kill cancerous B cells that cause B‐ALL. However, many patients experience a relapse of B‐ALL after CAR‐T therapy because a small population of residual leukemia cells remain in the bone marrow, where CAR‐T cells have limited trafficking and persistence. Modifying CAR‐T cells with surface receptors that enable them to invade the bone marrow has been effective in other leukemias.

In this study, Mao et al. induced anti‐B‐cell CAR‐T cells to produce excessive amounts of CXCR4, a receptor protein that helps guide cells to the bone marrow, which leukemia cells use to relocate to the bone marrow. They produced two kinds of modified CAR‐T cells, one that produced excess wild‐type CXCR4 (CXCR4^WT^) and another that produced excess CXCR4 with a S338X mutation (CXCR4^S338X^). The S338X mutation prevents the loss of CXCR4 receptors when cells enter and interact with surface marker proteins in the bone marrow niche.

In vitro tests showed that CXCR4 overexpression did not reduce these cells' ability to kill leukemia cells. The authors then induced human B‐ALL in immunodeficient mice. These mice were then injected with one of three types of anti‐B‐cell CAR‐T cells (unmodified, CXCR4^WT^, and CXCR4^S338X^). They found that CXCR4^WT^ and CXCR4^S338X^ migrated to the bone marrow in much greater numbers than unmodified cells.

They also found that CXCR4^S338X^ CAR‐T cells persisted in the bone marrow more effectively and showed stronger immune activity against B‐ALL than CXCR4^WT^ CAR‐T cells. Mice that received CXCR4^S338X^ CAR‐T cells were more likely to remain free of B‐ALL and survived for longer than mice that received unmodified or CXCR4^WT^.

These findings indicate that inducing anti‐B cell CAR‐T cells to overexpress CXCR4^S338X^ could improve the efficacy of treatment against B‐ALL and may support leukemia‐free survival, potentially reducing the need for hematopoietic stem cell transplants.

https://onlinelibrary.wiley.com/doi/full/10.1111/cas.70313

## Targeting SPP1‐CD44‐Hedgehog Axis Elicits Therapeutic Effects in Hepatocellular Carcinoma by Suppressing Intratumoral Fibrosis

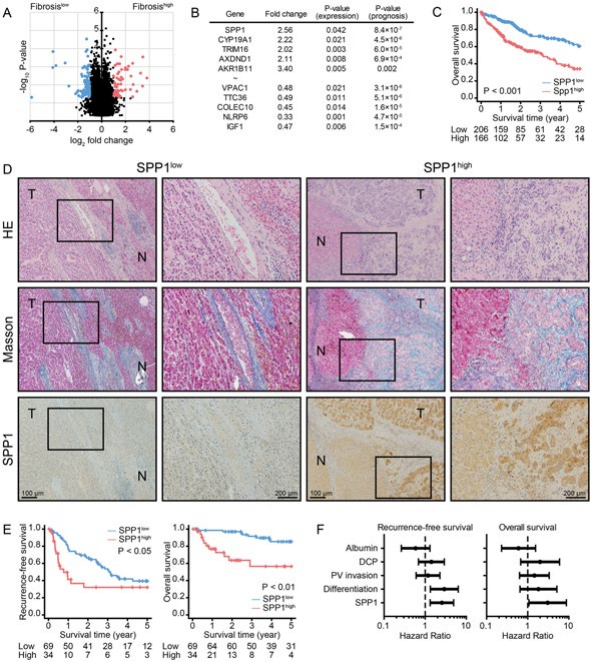

Hepatocellular carcinoma (HCC) often develops in fibrotic livers, where excess scar tissue not only impairs liver function but also creates an environment that promotes tumor growth and spread. Addressing this biological process has been a longstanding challenge in HCC treatment.

This study highlights a secretory protein called SPP1 as a major driver of liver fibrosis and tumor progression. The researchers studied mRNA and protein expression in HCC tissue samples and found that increased SPP1 levels were closely associated with intrahepatic and intratumoral fibrosis, as well as poor patient prognosis. They revealed that cancer cells with high SPP1 levels activate nearby hepatic stellate cells (HSCs), the main cells responsible for producing scar tissue. Laboratory and animal experiments demonstrated that this interaction stimulates the Hedgehog signaling pathway through the SPP1‐CD44 axis. Liver cancer cells producing more SPP1 caused more fibrosis and grew faster, while blocking the Hedgehog pathway using the drug vismodegib reduced scar formation and slowed tumor progression.

Together, these results suggest that SPP1 could serve as both a biomarker for identifying high‐risk patients and a therapeutic target by disrupting the harmful communication between tumor cells and HSCs. Targeting the SPP1‐CD44‐Hedgehog pathway offers a promising approach to improve outcomes for patients with fibrotic HCC.

https://onlinelibrary.wiley.com/doi/full/10.1111/cas.70296

## Urinary Tumor DNA to Identify Candidates for Repeat Transurethral Resection in Non‐Muscle‐Invasive Bladder Cancer

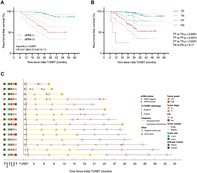

Non‐muscle invasive bladder cancer (NMIBC) is the most common form of bladder cancer at diagnosis. Since NMIBC recurs in many patients, standard monitoring protocols recommend repeat transurethral resection of bladder tumors (re‐TURBT) for high‐risk patients. However, more than half of patients who undergo re‐TURBT are ultimately found to have no remaining tumor. As a result, many patients may be exposed to an invasive and uncomfortable procedure without a clear benefit.

In this study, Xue and colleagues analyzed urine samples from 161 high‐risk NMIBC patients who were scheduled for re‐TURBT following their initial surgery. The researchers used a low‐coverage whole‐genome sequencing (LC‐WGS)–based multidimensional utDNA assay (utLIFE) to detect tumor‐derived DNA in urine by analyzing cancer‐related genetic changes. For comparison, they also performed standard urine cytology, which looks for cancer cells shed into the urine.

At re‐TURBT, residual cancer was detected in 57 patients (35%). The utDNA test identified 50 patients (31%) as positive for tumor DNA. Of these, 46 were confirmed to have residual cancer at re‐TURBT. Among the 111 patients with a negative utDNA result, only 11 had residual cancer. Patients with a positive utDNA result were approximately 77 times more likely to have residual disease than those with a negative result. Importantly, utDNA was a much stronger predictor of residual cancer than urine cytology.

Long‐term follow‐up showed that a positive utDNA result was also the strongest predictor of bladder cancer recurrence after re‐TURBT. It outperformed urine cytology as well as traditional clinical factors such as tumor stage, tumor grade, tumor size, number of tumors, and treatment type. In many cases, tumor DNA became detectable in urine months before recurrence was visible during cystoscopy.

Overall, these findings demonstrate that analyzing tumor DNA in urine provides a highly accurate, noninvasive way to detect remaining cancer and to predict future recurrence. The utDNA test has the potential to help doctors individualize postoperative care, avoid unnecessary repeat surgeries, and improve long‐term management of patients with NMIBC.

https://onlinelibrary.wiley.com/doi/full/10.1111/cas.70311

